# Coronary Artery-Left Ventricle Fistula: A Case Report of a Rare Connection Error!

**DOI:** 10.7759/cureus.266

**Published:** 2015-04-23

**Authors:** Dipti Sagar, Andres Hernandez, Todd Heimowitz

**Affiliations:** 1 Medicine, Albert Einstein College of Medicine; 2 Medicine, North University Hospital, Barranquilla, Colombia; 3 Interventional Cardiology, Mount Sinai Medical Center, Miami Beach, Florida,

**Keywords:** coronary artery fistula, left ventricle fistula, asymptomatic coronary fistula

## Abstract

Coronary artery fistulas (CAF) terminating in the left ventricle are rare. We present an asymptomatic 58-year-old African-American female with a unique fistula network detected during cardiac catheterization. Large coronary fistulas can have a silent presentation and may be an incidental finding not requiring intervention.

## Introduction

Coronary artery anomalies include anomalies of origin, termination, structure, or course. Coronary artery fistulae (CAF) are classified as abnormalities of termination and are considered a major congenital anomaly. Coronary artery fistula is defined as a connection between one or more of the coronary arteries and a cardiac chamber, or great vessel [1]. First described in 1865, approximately 55% of the cases involve the right coronary artery or its branches as the site of fistula origin while 35% originate from the left coronary artery. Over 90% of the fistulas drain into the right side of the heart with only 3% terminating in the left ventricle [2].

Coronary fistula communications can be congenital and acquired. Congenital coronary artery fistulae may occur as an isolated finding or may appear in the context of other congenital cardiac anomalies or structural heart defects. This most frequently occurs in critical pulmonary stenosis or atresia with an intact interventricular septum and in pulmonary artery branch stenosis, tetralogy of Fallot, coarctation of the aorta, hypoplastic left heart syndrome, and aortic atresia. Acquired coronary artery fistula may rarely arise as a consequence of a trauma, such as a gunshot wound or a stab wound. They can also occur after cardiac surgery or invasive cardiac catheterization with percutaneous transluminal coronary angioplasty, pacemaker implantation, or endomyocardial biopsy.

The factors that determine the hemodynamic significance of the fistulous connection include the size of the communication, the resistance of the recipient chamber, and the potential for development of myocardial ischemia. Occasionally, high-output congestive heart failure has been described. We present an interesting case of a 58-year-old female with a large coronary artery fistula terminating in the left ventricle, illustrating a unique fistula network.

## Case presentation

The patient is a 58-year-old African-American female who was admitted for abdominal pain for one week. She was found to have an abdominal incisional hernia. Past medical history included multiple hospitalizations for chest discomfort, diabetes mellitus, hypertension, gastroesophageal reflux disease, chronic obstructive pulmonary disease, and schizophrenia. Family history was significant for Type II diabetes mellitus and gastrointestinal bleed in the father and myocardial ischemia in the mother. There was no history of connective tissue disease or congenital heart disease in her family. Social history was remarkable for one pack per day of cigarette smoking and cocaine use. Informed patient consent was obtained prior to treatment.

On physical examination, her vitals were T 96.8, heart rate (HR) 94, RR 18, and supine left arm BP 134/81. The right arm BP was 134/84 mm Hg. The neck was supple with no carotid bruit or thyromegaly. Her cardiovascular exam was significant for an I-II/VI systolic murmur heard at the apex. S1/S2 were normal with regular rate and rhythm. There were no S3, S4 rubs or gallop. 

Pertinent lab values revealed thrombocytopenia (74,000 per microliter) and three sets of normal cardiac enzymes. A 12-lead electrocardiogram showed a sinus rhythm at the rate of 68, left axis deviation, T-wave inversion in V3-V6 with ST depression in V3-V6, and QTc 459 m sec. Dobutamine stress echocardiography was done in view of significant past medical history. A heart rate of 69 and an episode of idioventricular rhythm was achieved. No ischemia was noted at the HR achieved. Chest x-ray showed a normal cardiac silhouette with mild tortuosity and calcification of the thoracic aorta. A two-dimensional echocardiogram showed a normal LV systolic function and an EF of 60-65%, moderate concentric left ventricular hypertrophy, and normal AR and RV systolic pressure as measured by Doppler.

In light of patient's multiple hospitalizations for chest discomfort, non-diagnostic suboptimal stress echocardiogram, and abnormal EKG, a cardiac catheterization was recommended for risk stratification and clearance for abdominal surgery.

**Figure 1:**
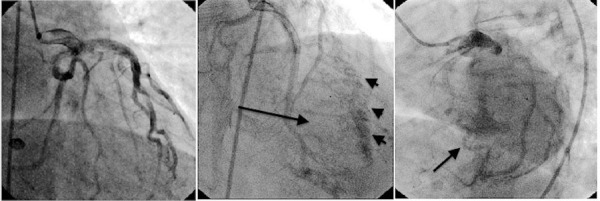
Cardiac catherization showing a large network of left coronary artery fistulas (arrows).

Left heart catheterization showed a widely patent but somewhat dilated left main coronary artery, as well as left anterior descending and left circumflex arteries with no significant focal obstruction. Of note, there was a large system coronary fistula arising mainly from the left circumflex coronary artery, which was terminating into the left ventricle through the lateral wall (Figure 1). The left ventricular end-diastolic pressure was 19 mm Hg with an ejection fraction of 60% with no significant valvular abnormalities or obstructive coronary artery disease.

In light of the patient's catheterization report, a diagnosis of a large left coronary fistula draining into the left ventricle was made. Retrospective studies have shown that such fistulas do not usually cause symptoms in the first two decades. Symptoms and complications, such as myocardial infarction, thrombosis, cardiac failure, atrial fibrillation, endocarditis, rupture, and arrhythmia, increase after this age [3]. Since the patient was asymptomatic and stable from a cardiovascular standpoint, she was assessed to be a low/intermediate risk candidate for abdominal surgery.

## Discussion

The majority of the coronary artery fistulas are congenital in origin, although they have been reported after cardiac surgery, such as valve replacement, coronary artery bypass grafting, and after repeated myocardial biopsies in cardiac transplantation [4]. Patients with a coronary artery fistula can present with symptoms of congestive heart failure (CHF), ischemia, and arrhythmias, or simply as an asymptomatic patient with a continuous murmur. If the fistula connects to the left ventricle only, an early diastolic murmur may be heard [3, 5].

Most coronary artery fistulas terminate in the right ventricle (90% of the cases), creating a left to right shunt, and thus overloading the pulmonary vasculature. Such patients may have a more symptomatic presentation. Over 90% of fistulas drain into the right side of the heart with only 3% terminating in the left ventricle. Coronary artery fistulas that drain into the left ventricle may hemodynamically compromise the myocardium, leading to myocardial ischemia. The pathophysiology involved is the "steal" phenomenon, which is the reduction of myocardial blood flow distal to the site of the fistula. This mechanism is related to the diastolic pressure gradient and run-off from the coronary vasculature to a low-pressure receiving cavity. If the fistula is large, the intracoronary diastolic pressure progressively diminishes [5].

The treatment for this abnormality can be preventive or curative. Surgical or interventional (coil placement) treatments are the options [5]. The decision to treat is based on the clinical presentation of the patient, age of diagnosis, and the hemodynamic significance of the fistula. Young individuals under 20 years of age should be treated for hemodynamically significant fistulas, irrespective of symptoms at the time of diagnosis, as three-quarters of patients presenting over the age of 40 have progressed to some level of heart failure [4].

Our patient had a giant left circumflex coronary artery fistula terminating in the lateral wall of the left ventricle that was hemodynamically insignificant with normal LV function, sustained ejection fraction, mildly increased end-diastolic pressure (may be due to hypertension), and absence of symptoms at the age of 58. Taking into consideration the aforementioned factors, we concluded that our patient would not benefit much from surgery.

## Conclusions

The importance of our case lies in the incidental finding of a rare left coronary fistula draining into the lateral wall of the left ventricle. This case also illustrates a rare benign presentation of a large network of left coronary fistulas that does not require any intervention.
